# Dynamic Disorder in Quasi-Equilibrium Enzymatic Systems

**DOI:** 10.1371/journal.pone.0012364

**Published:** 2010-08-24

**Authors:** Srabanti Chaudhury, Oleg A. Igoshin

**Affiliations:** Department of Bioengineering, Rice University, Houston, Texas, United States of America; Memorial Sloan Kettering Cancer Center, United States of America

## Abstract

Conformations and catalytic rates of enzymes fluctuate over a wide range of timescales. Despite these fluctuations, there exist some limiting cases in which the enzymatic catalytic rate follows the macroscopic rate equation such as the Michaelis-Menten law. In this paper we investigate the applicability of macroscopic rate laws for fluctuating enzyme systems in which catalytic transitions are slower than ligand binding-dissociation reactions. In this quasi-equilibrium limit, for an arbitrary reaction scheme we show that the catalytic rate has the same dependence on ligand concentrations as obtained from mass-action kinetics even in the presence of slow conformational fluctuations. These results indicate that the timescale of conformational dynamics – no matter how slow – will not affect the enzymatic rate in quasi-equilibrium limit. Our numerical results for two enzyme-catalyzed reaction schemes involving multiple substrates and inhibitors further support our general theory.

## Introduction

Enzymes are biomolecules that catalyze (i.e., increase the rates of) biochemical reactions. Kinetics of enzymatically controlled reactions is generally influenced by a variety of factors such as temperature, pH, ionic strength as well as concentrations of enzymes and ligands (substrates, products, inhibitors or activators) [1]. The dependence of enzymatically controlled reaction rate on these concentrations is often referred to as the *kinetic law*. The kinetic law can be deduced under certain approximation for an enzyme with known kinetic mechanism that is when we know how ligands bind and dissociate from the enzyme and at which state catalytic transitions occur. These kinetic laws have been derived for a variety of reaction schemes with mass-action kinetics and form a solid mathematical foundation of enzymology allowing the researchers to predict the kinetic laws from enzymatic mechanisms and *vice versa*. Ref. [1] gives a wide collection of examples of such complex enzyme catalyzed reaction mechanisms.

The simplest kinetic mechanism for enzymatic reaction with a single substrate assumes that the enzyme *E* combines with a substrate *S* to form the *ES* complex which undergoes irreversible reaction to form the product *P* and the original enzyme.
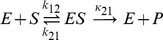
(1)The kinetic law for this reaction describing the rate of product formation, *V*, as a function of substrate concentration, [*S*], is given by the Michaelis-Menten(MM) equation [1], [2], [3]

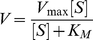
(2)This law is generally derived under the quasi-steady state assumption [1], i.e. when concentration of the substrate-enzyme complex, [ES], changes much slower than that of the product, [P]. The kinetic parameters of this kinetic law are the MM constant 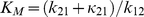 and maximal rate 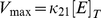 proportional to total enzyme concentration. For more complicated reaction schemes involving multiple ligands, multi-subunit enzymes and other complications the resulting kinetic laws are more complex but still can be derived following the standard procedures if the reaction mechanism is known and mass-action kinetics is assumed.

Results of recent single-molecule experiments shade some doubt on the applicability of simple mass-action kinetics to enzymatic reactions. Several experiments have shown that the catalytic activity of an enzyme fluctuates over a wide range of timescales [4], [5] (10^−4^–10s). These results illustrate a more general phenomenon termed a dynamic disorder [6], [7] – fluctuations of chemical reaction rates which originates as a consequence of slow conformational fluctuations that occur on similar range of timescales [8], [9], [10], [11]. Such experimental observations have inspired many theoretical studies [11], [12], [13], [14], [15], [16] investigating the effects of conformational fluctuations on the enzyme kinetic laws. The results of some of theoretical studies indicate that in general the steady state kinetic law for a fluctuating enzyme following the mechanism outlined in equation (1) is not of Michaelis-Menten form [11], [14]. However, there are several limiting cases in which the MM equation is obeyed even for single-molecule reactions: the *quasi-static* limit when the conformational dynamics in either *E* or *ES* state is much slower than in the other and the *quasi-equilibrium* limit when the catalysis is much slower than substrate dissociation reaction [14] (this limit is called rapid equilibrium in Ref. [1]). Both limits will result in the steady-state velocity for the reaction scheme (equation (1)) to be of the same form as macroscopic kinetic law of equation (2).

The above results bring two important questions: (1) whether the macroscopic kinetic laws hold in quasi-static or quasi-equilibrium limit for more complicated reactions schemes despite conformation fluctuations and (2) what kind of deviations one can expect when MM law breaks down. We have partially addressed these questions in our recent work [17] where we considered a kinetic scheme that explicitly includes product-release step,
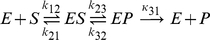
(3)We have shown that even in quasi-static limit the resulting kinetics deviates from those predicted by macroscopic kinetic laws and resulted in substrate inhibition effect. Moreover, this effect can under certain conditions lead to bistability in the reaction network. Our results thus indicated that conformational fluctuations in the enzymatic scheme with more than two states of the enzyme (*E*, *ES* and *EP* for equation (3)) will not generally result in macroscopic kinetic law in the quasi-static limit. The goal of this work is to investigate the general applicability of macroscopic rate laws for fluctuating enzyme systems in the quasi-equilibrium limit.

In an earlier work by Min et al [14] it was showed that the for a simple enzyme catalyzed reaction (1), the classical MM mass action kinetics is preserved in the quasi-equilibrium limit even in the presence of conformational fluctuations which are slower or comparable to other binding- dissociation processes. This suggested that the timescales of conformational fluctuations have no effect on the catalytic rate in the quasi-equilibrium limit. In this paper, we present a theory of the kinetics for fluctuating enzymes for an arbitrary reaction scheme – with a possibility of multiple substrates and cofactors allosterically modulating reaction rate. This work will therefore extend the results of Min et al [14] from a particular scheme corresponding to MM kinetics (kinetic scheme (1)) to a more general catalytic mechanism of arbitrary complexity [1].

The outline of the paper is as follows. In the **Methods** section we first present our notation and outline standard chemical-kinetics (mass-action) approaches to derive enzymatic rate laws for arbitrary reaction schemes in the steady state and in the quasi-equilibrium limits. We then introduce formalism to account for possibility of conformational dynamics of the free enzyme and its complexes. In the **Results and Discussion** section we analyze the kinetic laws resulting in the quasi-equilibrium limit and show that despite slow conformational fluctuations, the catalytic rate has the same dependence on substrate/modulator concentration as obtained from conventional mass action kinetics. We further support our general theory using two complex enzyme catalyzed reaction schemes involving multiple substrates and inhibitors and use numerical simulations to test our analytical predictions and show the nature of possible deviations from macroscopic rate laws.

## Methods

### Mass-action approaches to enzymatic kinetic laws

#### General reaction scheme and kinetic equations

**Figure 1:**
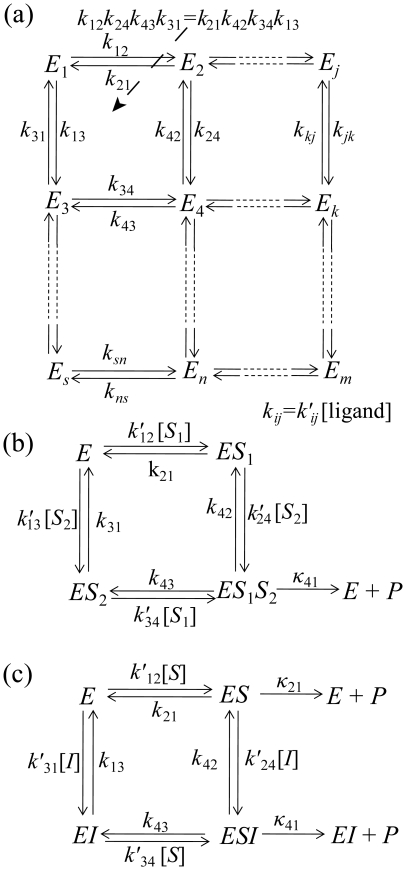
Reaction schemes under consideration. (a) Schematic diagram of the transitions between various states of enzyme for an arbitrary reaction scheme discussed in Methods. The detailed balance condition is shown for the loop comprised by states 1—4. For simplicity only reversible transitions are shown and catalytic transitions are omitted. The rate constants for ligand-binding reactions are proportional to concentrations as indicated. (b) Reaction scheme for random order bisubstrate reaction. (c) Reaction scheme for partial noncompetitive inhibition.

To illustrate our notation and lay grounds for subsequent formalism involving conformational dynamics of the enzyme, we begin with presenting a classical mass-action framework used to obtain rate-law for an arbitrary reaction scheme. To help the reader better grasp the notation we present particular examples of this formalism in the Section A of Text S1. Consider an enzyme (Fig. 1a) that can be present in *N* different states, *E_i_*, corresponding to free-enzyme state and various complexes with substrates, inhibitors, activators etc. Reversible transitions between these states – binding and dissociation reactions – are represented as effective first-order reactions
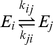
(4)where *k_ij_* and *k_ji_* are the forward and backward transition rates respectively. For ligand-binding reactions, forward rate is a function of ligand concentration (e.g. in equation (1), for the *E→ES* transition, the rate will be *k_12_*[S]). Importantly, we assume that reactions represented in equation (4) are truly reversible and do not consume energy (e.g. do not result in ATP hydrolysis). Therefore, the rate constants of these reactions are generally not mutually independent and are subject to detailed balance constraints (see below). On the other hand, catalytic transitions between states are usually associated with large free energy drops supplied by “energy-currency” biochemical molecules such as ATP. For notation simplicity, here we consider these to be irreversible given by a set of transitions
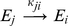
(5)Kinetics of the reaction scheme (equations (4) and (5)) are given by the set of differential equations of the conventional mass-action kinetics,
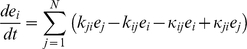
(6)Here we introduce the normalized concentrations or probabilities of finding enzyme in each state as 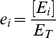 with *E_T_* being the total concentration of the enzyme, 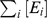.

The first two terms in equation (6) takes care of the reversible transitions not involved in catalysis and the remaining two terms are the catalytic terms. These equations are linearly dependent and need normalization condition for a unique solution:
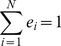
(7)


#### Steady-State and Equilibrium Rate Law

In the most common experimental setup the concentrations of enzyme molecules are significantly less than those of the ligands and, therefore, the concentration of ligands may not change significantly on the timescale the distribution of enzyme species reach steady-state. Therefore we assume that the concentration of the substrate or any other ligand in this arbitrary reaction scheme remains constant in time. This is analogous to the reactant steady approximation (RSA) as proposed by Hanson and Schnell [18]. According to their theory, the RSA and quasi-steady state approximation [19], [20] are two different approximations and there are instances where the quasi-steady state approximation can be valid without the RSA. Establishing criteria for comparing validity of quasi-steady state approximation and RSA for the reacting system with slow conformational fluctuations of the enzyme is potentially interesting; however, it is not dealt in here.

Thus in the steady state approach [1], [3] it is assumed that the enzyme species complex attains a nearly constant concentration within a short time after starting the reaction, i.e. the rate of change in the concentration of the enzyme-species complex is equal zero. Within the steady state approximation, the LHS of equation (6) is zero, and we have
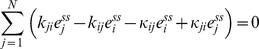
(8)where *ss* superscript defines steady-state enzyme state probabilities. For an enzyme in *N* different states, there are *N* linearly dependent equations. The equation for the free enzyme can be excluded leading to *N*-1 linearly independent equations. Together with the constraint given in equation (7) these linear equations can be solved for the probabilities 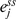. The steady state enzymatic rate (per unit of enzyme concentration) is given by
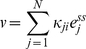
(9)For the kinetic scheme in equation (1), the quasi-steady state approximation (rate of change of [*ES*] is equal to zero) leads to MM law (equation (2)) with 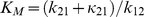.

On the other hand, the quasi-equilibrium limit assumes the distribution of various enzyme complexes quickly reaches equilibrium. This is a stronger requirement than steady state assumption and it is only valid when the catalytic rates (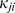) are much slower than the rate at which any enzyme-ligand complex dissociation (*k_ji_*). In this condition, the enzyme reaches equilibrium between its forms prior to a catalytic transition. In the quasi-equilibrium limit, equation (8) reduces to
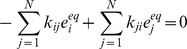
(10)However, these equations are simplified much more if we assume transitions characterized by *k_ij_* to be truly reversible, i.e. only involve ligand binding and dissociation reactions and do not consume external energy. In this case the binding-dissociation rates obey the detailed balance condition which states that for any closed loop in a reaction diagram, the product of the transition rates in the clockwise direction is same as the product of the transition rates in the anti-clockwise direction (cf. Fig. 1a).
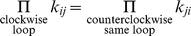
(11)These conditions ensure that in the absence of catalytic transitions the reactions reach true equilibrium in which not only the sum of all reaction fluxes for each enzyme pull (Equation (10)) but also individual fluxes for each irreversible 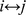 reaction are equal to zero. As a result, simplified equilibrium equations can be used to solve for equilibrium probabilities:
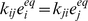
(12)The matrix resulting from equations (10) and (12) contains fewer non-zero elements and therefore often results in less complicated expressions for equilibrium rate-equation given by
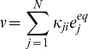
(13)For kinetic scheme in equation (1), the quasi-equilibrium limit also leads to MM law (equation (2)) but with equilibrium dissociation constant as MM-constant, i.e. 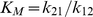.

Note that in equation (13), catalytic rates 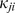 generally do not depend on concentrations so the concentration-dependence of rate comes through probabilities 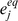. For illustration of the formalism discussed in this section, we derive the mass-action kinetic laws for noncompetitive inhibition mechanism in the Section A of the Text S1.

### Conformational dynamics in enzymatic kinetics

To investigate the effects of conformational fluctuations on the steady state kinetics of a fluctuating enzyme we introduce a continuous conformational coordinate *x*, characterizing enzyme and its complexes [14], [16]. For notation simplicity we assume this coordinate to be one-dimensional (one of the degrees of freedom is rate limiting) but all our results are straightforwardly generalizable for multi-dimensional conformational space. Continuous treatment of the conformational coordinates is the most general formalism but often these can be approximated into discrete state models [11], [12], [13]. These simple discretized models can lead to closed-form solutions for single-molecule enzymatic kinetics and are often in good agreement with continuous description. However some recent results on the measurement of the fluctuation dynamics show that the fluctuations occur on a wide spectrum of time-scales [4], [10], [11] thereby suggesting that a continuous treatment of the conformational coordinate with a Smoluchowski-Fokker-Plank equation is a more reasonable description than a few different conformational states. To account for enzyme conformational fluctuations for an arbitrary scheme of enzyme catalyzed reaction (equations (4) and (5)) one has to analyze coupled reaction diffusion equations [14], [21] for evolution of the probability of finding the enzyme in the state *i* at a conformational coordinate *x* at time *t*, 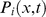


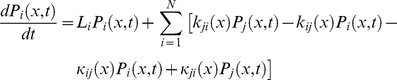
(14)Here the Smoluchowski operator *L_i_* is given by
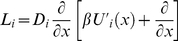
(15)where the prime sign denotes the first derivative over *x*; *D_i_* is the diffusion coefficient and *U_i_* is the potential energy landscape along conformational coordinate of the state *i* of the enzyme. Since this potential energy is defined up to a constant, and therefore without loss of generality we choose potentials so that:
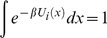
(16)As before, we are interested in the reaction rate after a stationary distribution of enzyme species and conformations have been reached. In the steady state (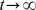 limit), the LHS of equation (14) reduces to zero and 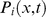 is replaced by its steady state probabilities, 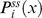 respectively.
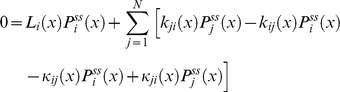
(17)Summing up these *N* coupled reaction diffusion equations leads to
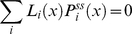
(18)Since the diffusion operator is conservative, that is 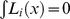, we note that the steady state distributions can be normalized as
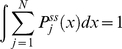
(19)Using equation (17) and (19) one can numerically solve for the steady state distributions. These distributions are then used to compute the steady state reaction rate (per molecule of enzyme)
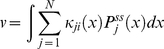
(20)This reaction rate is a function of the concentrations of substrates, inhibitors, activators and other possible cofactors modulating enzyme activity. These concentrations enter equation (17) through rates *k_ij_* and, therefore, affect probability 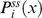. In general, the steady-state velocity from equation (20) has different ligand-concentration dependence than that given by mass action kinetics (cf. equation (9)). For instance, for two-state scheme with single-substrate reaction, equation (1), the rate expression for the fluctuation enzyme does not have MM form. However, as we show in the next section, conformational fluctuations do not affect the concentration dependence of the kinetic law in the quasi-equilibrium limit. The derivation in the next section is general and should apply to any arbitrary reaction scheme. The derivations for two particular reaction schemes (Fig. 1b and 1c) are sketched in the Section B of Text S1 as examples to help reader with the notation.

## Results and Discussion

### Quasi-equilibrium kinetic laws

#### Decoupling ansatz

For the reaction scheme in equation (1), Gopich and Szabo [14] have shown that the general formalism to monitor catalytic turnover events can be simplified if the conformational dynamics is much slower compared to substrate binding and catalytic reactions. In that case reaction-transition and diffusion probabilities can be decoupled. For our generalized reaction scheme this decoupling will result in the following ansatz solution for equation (17)
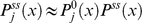
(21)where 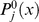 is the steady state probability of each state *j* of the enzyme for a fixed value of the conformational coordinate *x*. It obeys equation (17) without diffusion terms(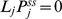) satisfies the condition
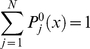
(22)It can be shown that this *ansatz is exact* in the quasi-equilibrium limit (cf. Section C of the Text S1).

Using the equation (21) in equation (18), we obtain the following equation for 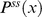


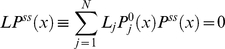
(23)where we introduce an linear operator *L* acting on 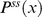. It is straightforward to show that operator *L* is effective diffusion operator of Smoluchowski form as in equation (15). Indeed, using equation (15) in equation (23) can be written as
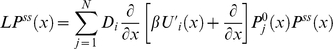
(24)Using the chain rule we obtain
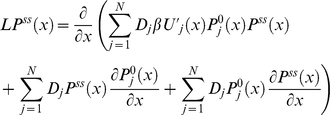
(25)Collecting the coefficients of 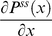 in equation (25), we define an effective diffusion coefficient
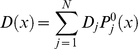
(26)Collecting the terms containing 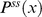 in equation (25), we introduce effective steady state potential *U_ss_(x)* as follows
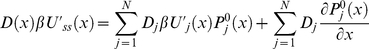
(27)Taking the derivative of equation (26) and using it in equation (27) we obtain after dividing by *D*(*x*)
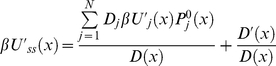
(28)Integrating both sides of equation (28) over *x*, we have
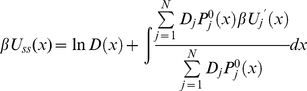
(29)As a result the effective diffusion operator *L* turns out to be the Smoluchowski operator with coordinate-dependent diffusion coefficient
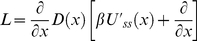
(30)with *D*(*x*) and *U_ss_*(*x*) defined by equation (26) and (29) respectively. Therefore, the general solution of equation (30) results in the steady state conformational distribution 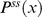 proportional Boltzmann distribution 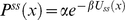. Using normalization of the steady state distributions 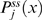 and 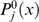 (equation (19) and (22)), we conclude 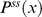 is normalized as 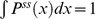. As a result we obtain the following solution for 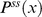


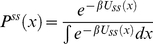
(31)Using equation (21) and (31), the steady state rate *v* in equation (20) reduces to
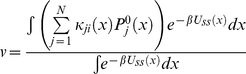
(32)


#### Quasi-equilibrium with detailed balance condition

In many enzymatic reactions, the catalytic rates 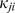 are slower than all other transition rates. This assumption is referred to as the quasi-equilibrium (or rapid quilibrium) condition. In this limit the steady-state probabilities satisfy the detailed balance condition assuring the fluxes of each individual reaction vanish for any arbitrary 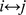 transition
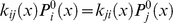
(33)As in mass-action kinetics (cf. Methods) these equations result in interdependencies of reaction rates (equation (11)). These conditions are satisfied for all *x* if one used transition-state expressions for reaction rates given by
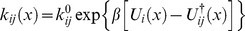
(34)where 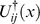 is the transition state potential and the prefactors 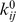 and 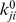 obey the condition (11), that is 
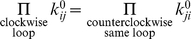
. These prefactors are independent of the conformational coordinate of the enzyme *x* but for bimolecular binding reactions they are functions of ligand concentrations.

Using equation (34) in equation (33) we get
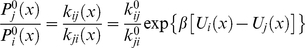
(35)Without loss of generality we can search for a solution for 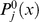 in the form of
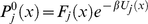
(36)Using equation (36), we obtain that 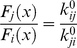. This relation implies that all *F_j_* must have the same conformation coordinate dependence, e.g,
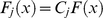
(37)where the constants *C_j_* are independent of *x*. Using equations (36) and (37) one therefore obtains the following solution for 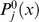 in the quasi-equilibrium limit
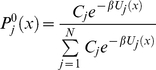
(38)Using equation (26) and (38) in equation (29) we have
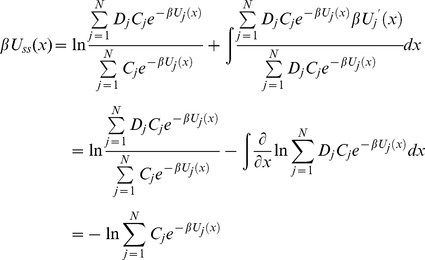
(39)Taking exponentials on both sides of the equation we conclude that
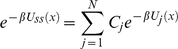
(40)With the use of equation (40) it can be shown that equation (32) reduces to
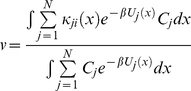
(41)Defining the conformational equilibrium average in the state *j* as 
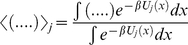
, the steady state velocity per molecule of the enzyme is given by
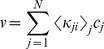
(42)where 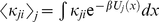 and
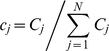
(43)Combining equation (35) and (38), and using the relation in equation (43) we find
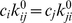
(44)


Comparing equation (12) and (44) we conclude that each *c_i_* is equal to probability of finding the enzyme in state *i*, *e_i_*, computed from conventional mass-action kinetics with rate prefactors 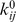 serving as a mass-action rate constants. Hence for any arbitrary enzyme catalyzed reaction the steady state velocity in the quasi-equilibrium limit has same dependence on substrate concentration as obtained from mass action kinetics with position-independent prefactors used as rates. One straightforward conclusion from this results states that in the quasi-equilibrium limit, the steady state reaction rate only depends on ligand concentrations and rate prefactors and *does not depend* the conformational diffusion coefficients. We illustrate this idea for two different reaction mechanisms (Fig. 1b, c) in next section and also show it assuming discrete conformational states in the Section D of the Text S1.

### Examples

We have shown in the previous section that even in the presence of slow conformational fluctuations, quasi-equilibrium condition results in the same dependence of the steady state enzymatic velocity on ligand concentration as observed in mass action kinetics. We have also concluded that the validity of this approximation does not depend on the timescale of the conformational dynamics. To support our general theory we consider two reaction schemes involving the binding of multiple ligands (substrates and inhibitors) to the free enzyme (Fig. 1b and c).

#### Bisubstrate random-order mechanism

Let us first consider a reaction scheme as shown in Fig. 1b which involves the random binding of two substrates *S_1_* and *S_2_* to the enzyme *E* followed by product formation. For this scheme the steady state rate of product formation per molecule of the enzyme (equation (20)) is given by:
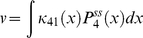
(45)where 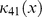 is the catalytic rate and 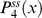 is the probability of finding the enzyme in the catalytic state (*ES_1_S_2_* form, state 4) at a conformational coordinate *x*. As shown in Section B of the Text S1, in the quasi-equilibrium limit, the steady state rate has the same dependence on the substrate concentration (equation (B.4)) as those obtained from mass action kinetics (equation (A.5)).

**Figure 2:**
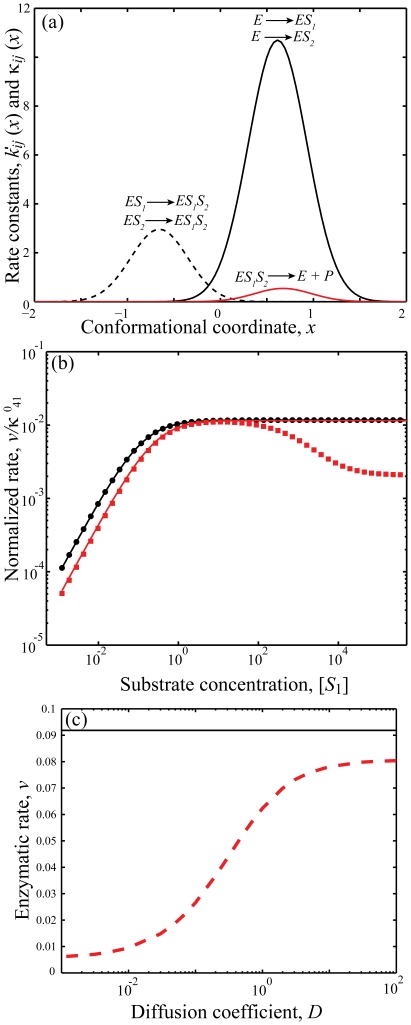
Numerical results for the random order bisubstrate reaction. (a) Rate constants *κ_ij_* (*x*) and *k_ij_* (*x*) as function of the conformational coordinate *x*, with parameters given in Tables 1 and 2 calculated from Eq. (34) and 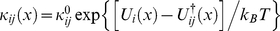. (b) Normalized rate 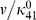 as a function of concentration [*S_1_*] at a fixed concentration of [*S_2_*] = 10. When catalytic reaction is fast with 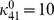, slow diffusion in *ES* conformation leads to non-monotonic dependence —substrate inhibition effect (red squares) and a deviation from the macroscopic rate law (red solid line) computed from mass action kinetics. For slow catalysis (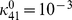, quasi-equilibrium limit) normalized rate 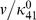 has the same dependence on *S_1_* concentration (black circles) as the macroscopic kinetic law (black solid line) calculated from equation (B.4). We use *D_ES1_* = 10^−2^ and *D_E_* = *D_ES2_* = *D_ES1ES2_* = 10^2^ and the rest of parameters as in (a). c) Enzymatic rate as a function of conformational diffusion. We took all the diffusion coefficients to be the same *D_E_* = *D_ES1_* = *D_ES2_* = *D_ES1S2_* = *D*. For fast catalysis 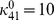 the enzymatic rate decreases with decreased diffusion (red dashed line). In the quasi-equilibrium limit, when the catalysis is slow 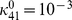 the enzymatic rate does not depend on the diffusion coefficient (black solid line). Notably the same trend continues with further decrease in diffusion coefficients, *D*. We use [*S_1_*] = [*S_2_*] = 1 and the remaining parameters as in (a, b).

To further verify these analytical results, to check the validity of the quasi-equilibrium approximation and to look at the effects of conformational dynamics in the general case we solve the coupled reaction diffusion equations for the reaction scheme(equation (B.2)) numerically using the Wang algorithm [21], [22] with transition rates given by equation (34). The catalytic rates 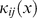 are same as shown in equation (34) with 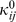 as the position-independent prefactor. The potentials *U_i_* (*x*) and 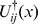 are modeled as harmonic potentials (we measure the potentials in the units of 1/β = *k_B_T* and drop this factor):
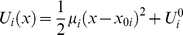
(46)and
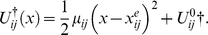
(47)The enzyme turnover rate can be calculated numerically using equation (45), (46) and (47) with parameters given in Tables 1 and 2. Fig. 2a shows the plot of the transition rates as functions of the enzyme conformational coordinate *x* for the parameters chosen calculated using equation (34); the same transition-state potential form is assumed for the catalytic rates. We assumed different conformational coordinates for maximum rate of initial substrate binding (*E*+*S_1_*→*ES_1_*, *E*+*S_2_*→*ES_2_*) as compared to subsequent binding(*ES_1_*+*S_2_*→*ES_1_S_2_*, *ES_2_*+*S_1_*→*ES_1_S_2_*).

**Table 1:** Model parameters for enzyme states for the reaction scheme in Fig. 1b.

	*E*	*ES_1_*	*ES_2_*	*ES_1_S_2_*
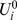	0	−1	−1	3
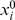	1	−0.5	−0.5	0
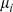	1	0.5	0.5	0.4

**Table 2:** Model parameters for transitions between different enzyme states for the reaction scheme in Fig. 1b.

	*E→ES_1_* *E→ES_2_*	*ES_1_→E* *ES_2_→E*	*ES_1_→ES_1_S_2_* *ES_2_→ES_1_S_2_*	*ES_1_S_2_→ES_1_* *ES_1_S_2_→ES_2_*	*ES_1_S_2_→E+P* (catalysis)
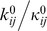	20	20	8	8	10
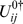	3	3	6	6	3
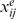	0.65	0.65	−0.65	−0.65	0.65
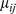	11.1	11.1	11.1	11.1	11.1

The results of the simulations are depicted in Fig. 2b which shows the steady state rate *v* as a function of [*S_1_*] with fixed [*S_2_*] when the conformational dynamics in the *ES_1_* state is very slow. For the case in which the catalytic rate is comparable to the dissociation rates of *S_1_* or *S_2_* from *ES_1_S_2_* state, slow conformational fluctuations have a significant effect on the kinetic law leading to non-monotonic dependence of *v* (red squares). This effect resembles substrate inhibition observed in our earlier work [17] for a different reaction scheme. The effect is not present in mass-action kinetics (red solid line). To understand the origin of substrate inhibition in this random-order bi-substrate reaction, one needs to focus on Fig. 2a, which indicates the ranges of conformational coordinates where transitions between the different states take place. The catalytic reaction *ES_1_S_2_*→*E*+*P* occurs along the positive values of *x* (rate 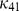, red solid line). The regenerated enzyme *E* then combines with the free substrate *S_1_* and *S_2_* to form the *ES_1_* and *ES_2_* complexes. The conversion from *ES_1_*→*ES_1_S_2_* and *ES_2_*→*ES_1_S_2_* takes place along the negative values of *x* (rates *k_34_* and *k_24_*, dashed line). At a fixed concentration of *S_2_*, and at high *S_1_* concentration, the free enzyme regenerated after the product release step (*ES_1_S_2_*→*E*+*P*) quickly binds to the substrate *S_1_* to form the *ES_1_* complex along the positive values of *x*. This *ES_1_* complex needs to relax and change its conformation into the negative region of *x* for the reaction *ES_1_*→*ES_1_S_2_* to take place. Since conformational dynamics in the *ES_1_* state is very slow, this order of substrate binding (*S_1_* first, then *S_2_*) would result in slower catalytic rate as compared to a different order (*S_2_* first, then *S_1_*). But as the substrate concentration *S_1_* is increased the probability of the substrate *S_2_* to bind first to the enzyme decreases essentially pushing the reaction to proceed through the *ES_1_* state and thereby leading to a decrease of the overall catalytic flux. Importantly, this effect does not happen in the quasi-equilibrium limit (when 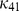 is very slow). In this limit the rate law is not only monotonic (black circles) but also coincides with the steady state rate as obtained from mass action kinetics (black solid line). Moreover, in agreement with our theoretical derivation, the enzymatic rate does not depend on the diffusion when the catalytic rate is slow (Fig. 2c).

#### Partial noncompetitive inhibition

We also consider another scheme described in Fig. 1c. In this enzyme catalyzed reaction, there are two catalytic reactions which lead to product formation, one from the *ES* complex and another from the *ESI* complex. The steady state rate of product formation is therefore given by
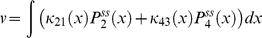
(48)where 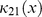 and 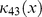 are the catalytic rates. 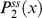 and 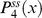 are the probabilities of finding the enzyme in the *ES* (state 2) and *ESI* form (state 4) respectively at a conformational coordinate *x*. When 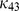
≪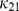, then the *ESI* complex cannot produce product as effectively as *ES* complex leading to decrease of catalytic rate as probability of *ESI* state formation increases at rising inhibitor concentrations.

**Figure 3:**
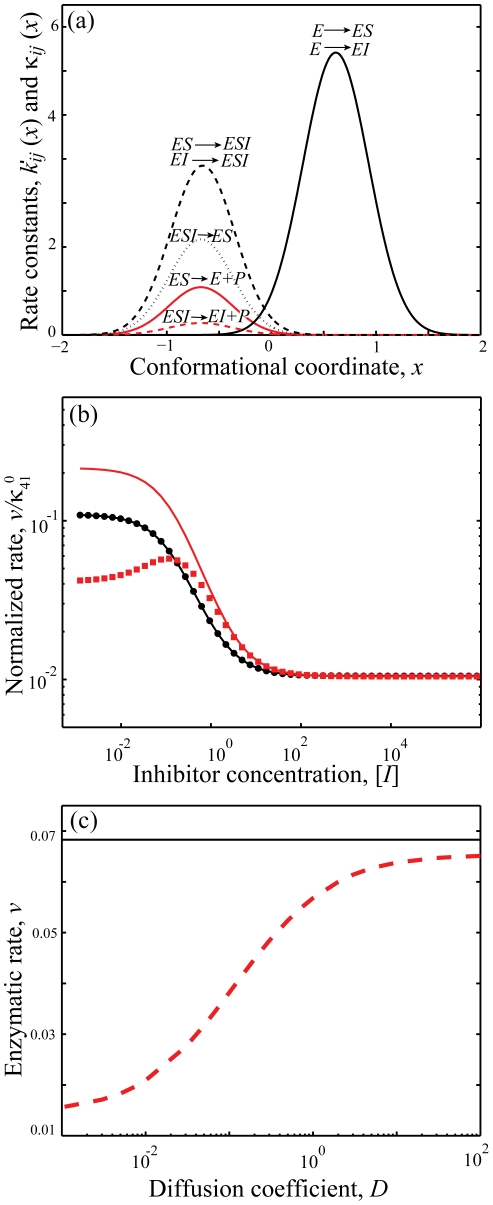
Numerical results for the partial noncompetitive inhibition reaction. a) Rate constants *κ_ij_* (*x*) and *k_ij_* (*x*) as function of the conformational coordinate *x*, with parameters given in Tables 3 and 4 calculated using Eq. (34) and 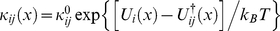. b) Normalized rate 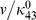 as a function of concentration [*I*] at a fixed concentration of [*S*] = 1. When catalytic reaction is fast with 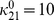 and 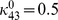 Slow diffusion in *ES* conformation leads to an increase in the rate at low and intermediate inhibitor concentration followed by a decay (red squares), a deviation from the macroscopic rate law(red solid line) computed from equation (A.3). For slow catalysis (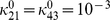, quasi-equilibrium limit) normalized rate 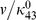 has the same dependence on *I* concentration (black circles) as the macroscopic rate law(black solid line) calculated from equation (B.7). We use *D_ES_* = 10^−2^ and *D_E_* = *D_EI_* =  = *D_ESI_* = 10^2^ and the rest of parameters as in (a). c) Enzymatic rate as a function of conformational diffusion. We took all the diffusions to be the same *D_E_* = *D_ES_* = *D_EI_* = *D_ESI_* = *D*. For fast catalysis 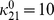 and 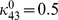 the enzymatic rate decreases with decreased diffusion (red dashed line). In the quasi-equilibrium limit, when the catalysis is slow 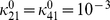 the enzymatic rate does not depend on the diffusion coefficient (black solid line). Notably the same trend continues with further decrease in diffusion coefficients, *D*. We use [*S*] = 1, [*I*] = 0.1 and the remaining parameters as in (a, b).

Fig. 3a shows the transition rates for Fig. 1c as a function of the conformational coordinate *x* calculated using equation (34); the same transition-state potential form is assumed for the catalytic rates. Fig. 3b is a plot of the normalized steady state rate calculated numerically using. equation (B.4) and (48) with the potentials as defined in equation (46) and (47) as a function of the inhibitor concentration [*I*] with the conformational dynamics in *ES* state is very slow. The parameter values for the numerical simulations are taken from Tables 3 and 4. For the case in which the catalytic rates are comparable to the dissociation rates of *S* or *EI* from the *ES* or *ESI* complex respectively, slow conformational fluctuations lead to an increase in the rate at low and intermediate *I* concentrations followed by decrease with the further increase in *I* concentration (red squares). To understand the origin of this effect, one needs to focus on Fig. 3a, which indicates the ranges of conformational coordinates where transitions between the different states take place. The faster catalytic reaction *ES*→*E*+*P* occurs along the negative values of *x* (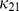: red solid line). But the binding of the enzyme *E* with the substrate *S* is more likely to occur along the positive values of *x* (rate *k_12_*, black solid line). Since conformational dynamics in the *ES* state is very slow, the flux through the pathway *E+S*→*ES*→*E*+*P* is limited by the conformational relaxation in *ES* state and, therefore, can be small. On the other hand, if inhibitor has a chance to bind to the enzyme first, another, faster catalytic pathway is possible, inhibitor binds first, then substrate binds, then inhibitor dissociates and catalysis occurs: *E+I*→*EI+S*→*ESI*→*ES+I*→*E+P+I*. Because the inhibitor is likely to dissociate in the negative conformation, slow conformational diffusion in *ES* state does not affect the flux. This second pathway becomes more likely as concentration of inhibitor, *I*, increases initially. This initial increase thereby leads to increase in overall catalytic rate. As inhibitor concentration further increased, *ES* state is more likely to bind the inhibitor, *I*, and the flux get limited by the catalytic rate from *ESI* state. This effect leads to decrease of the enzymatic rate at higher inhibitor concentrations toward smaller catalytic rate 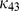 (red dashed line in Fig. 2a). The above described effect does not play a role in the quasi-equilibrium limit (when 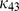 and 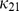 are smaller than *k_ij_*). In this limit the rate law is not only monotonic (black circles) but also coincides with the steady state rate as obtained from mass action kinetics (black solid line). Intuitively, this occurs because in the equilibrium the flux does not depend on the pathway. In addition, as suggested by our theoretical derivation, the enzymatic rate does not depend on the diffusion when the catalytic rate is slow (Fig. 3c).

**Table 3:** Model parameters for enzyme states for the reaction scheme in Fig. 1c.

	*E*	*ES*	*EI*	*ESI*
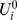	0	−1	−1	3
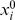	1	−0.5	−0.5	0
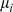	1	0.5	0.5	0.4

**Table 4:** Model parameters for transitions between different enzyme states for the reaction scheme in Fig. 1c.

	*E→ES* *E→EI*	*ES→E* *EI→E*	*ES→ESI* *EI→ESI*	*ESI→ES* *ESI→EI*	*ES→E+P* (catalysis) *sstep)*	*ESI→EI+P* (catalysis)
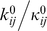	20	20	8	8	10	0.5
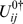	3	3	6	6	3	3
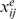	0.65	0.65	−0.65	−0.65	−0.65	−0.65
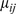	11.1	11.1	11.1	11.1	11.1	11.1

### Concluding remarks

In this paper we developed a generalized formalism to study the kinetics of an enzyme with arbitrary complicated kinetic mechanism in the presence of dynamic disorder. Slow conformational fluctuations which are a source of dynamic disorder are common to many enzymes and can lead to deviations from macroscopic rate laws as predicted by conventional chemical kinetics. Here we have focused on the kinetic laws in the quasi-equilibrium limit where catalytic transitions are slower than ligand binding-dissociation reactions. Our results indicate that even in the presence of slow conformational fluctuations macroscopic rate laws will hold in this limit. This implies that the steady state rate has the same dependence on the ligand concentration as observed in conventional mass action kinetics for any arbitrary enzyme catalyzed reaction network. This dependence will coincide with that obtained from conventional mass-action kinetics using conformation-independent rate prefactors as rate constants. This result extends the previous work of Min *et al.* [15] from simple Michaelis-Menten scheme to a kinetic scheme of arbitrary complexity with multiple substrates and allosteric ligands. As a consequence, in this quasi-equilibrium limit, the rate no longer depends on the conformational dynamics of the enzyme (Fig. 2c and Fig. 3c). Our analytical predictions are further supported by numerical simulations for the two complex reaction schemes (Fig. 1bc). Importantly these simulations also indicate that quasi-equilibrium limit can be achieved when conformational dynamics is very slow, even when it is slower than the catalytic rate. The obtained conclusions are therefore applicable to any enzyme with arbitrary complex kinetic mechanism (multiple substrates, cofactors, allosteric ligands) as long as the catalytic steps are slower than ligand dissociation reactions.

In the single molecule enzyme experiment on the catalytic activity on the enzyme 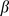-galactosidase the MM behavior of the average number of catalytic turnovers per unit time still holds [4] even in the presence of fluctuations on all time scales. The quasi-equilibrium condition provides a plausible explanation of this effect [14]. How general is quasi-equilibrium limit for enzymatic kinetic systems with possibly slow fluctuating enzyme conformations? One can argue that for some enzymatic reactions where the substrate specificity actually comes about from different binding affinities rather than different catalytic rates. In that case for efficient specificity selection, multiple binding-dissociation must to occur before the catalysis to proceed. As a result, these enzymes will essentially operate in quasi-equilibrium limit. On the other hand, some of enzymes have evolved to function in catalytically perfect regime, i.e. when the catalytic rate is much faster than substrate dissociation [23], [24]. These enzymes do not operate in quasi-equilibrium and dynamic disorder may affect their kinetic laws. However, it is not clear how common such enzymes are, given that fast catalytic efficiency does not always directly transfer into fitness [25]. In any case, comparison of the turnover rate of the reaction at low substrate concentration to that predicted by diffusion-reaction theory can aid in predicting if the enzyme operates near quasi-equilibrium limit.

A classical model of allosteric regulation assume that binding of small-molecule regulator into some distant, non-catalytic site affects the reactions in the catalytic site and therefore changes the catalytic flux. Recently, Xing proposed [21] that the slow conformational dynamics of allosteric proteins is a possible alternative to allosteric regulation mechanism. In this scheme, binding of ligand leads to an increased roughness of potential-energy landscape and thereby affect the enzymatic rate through conformational diffusion. Our theoretical results place an important constrain for such non-allosteric regulation mechanism to be significant. We show that the timescale of conformational transitions (no matter how slow) does not affect the reaction flux if the catalytic rates are much slower than ligand dissociation rates (quasi-equilibrium limit). Thus dynamic disorder may only affect the kinetic laws when catalytic transitions are fast.

## Supporting Information

Text S1 (A) Mass-action kinetics for random-order bisubstrate and partial noncompetitive inhibition reaction schemes. (B) Conformational dynamics for random-order bisubstrate and partial noncompetitive inhibition reactions. (C) Decoupling ansatz is exact in quasi-equilibrium limit. (D) Discrete-state model for conformational fluctuations in the quasi-equilibrium limit.
(0.10 MB PDF)
