# Supplementary material for: Dynamic Disorder in Quasi-Equilibrium Enzymatic Systems
Source: PLoS One. 2010 Aug 24;5(8):e12364. doi: 10.1371/journal.pone.0012364 (PMC2927515; doi:10.1371/journal.pone.0012364)
Supplement: Text S1 — (A) Mass-action kinetics for random-order bisubstrate and partial noncompetitive inhibition reaction schemes. (B) Conformational dynamics for random-order bisubstrate and partial noncompetitive inhibition reactions. (C) Decoupling ansatz is exact in quasi-equilibrium limit. (D) Discrete-state model for conformational fluctuations in the quasi-equilibrium limit. (0.10 MB PDF) [file pone.0012364.s001.pdf]

SUPPORTING INFORMATION

A: Mass-action kinetics for random-order bisubstrate and partial noncompetitive inhibition reaction schemes

Let's consider the scheme shown in Fig. 1c. Conventional mass action kinetics results in the following differential equations (equation (6)) for the normalized concentrations (probabilities) of various enzyme forms in this scheme:

$$\frac{de_1}{dt} = -\left(k_{12}[S]+k_{13}[I]\right)e_1 + k_{21}e_2 + k_{31}e_3 + \kappa_{21}e_2 \qquad \text{(A.1a)}$$

$$\frac{de_2}{dt} = -\left(k_{21}+k_{24}[I]\right)e_2 + k_{42}e_4 + k_{12}[S]e_1 - \kappa_{21}e_2 \qquad \text{(A.1b)}$$

$$\frac{de_3}{dt} = -\left(k_{34}[S]+k_{31}\right)e_3 + k_{13}[I]e_1 + k_{43}e_4 + \kappa_{43}e_4 \qquad \text{(A.1c)}$$

$$\frac{de_4}{dt} = -\left(k_{43}+k_{42}\right)e_4 + k_{24}[I]e_2 + k_{34}[S]e_3 - \kappa_{43}e_4 \qquad \text{(A.1d)}$$

The steady state velocity is given by

$$v = \kappa_{21}e_2^{ss} + \kappa_{43}e_4^{ss} \qquad \text{(A.2)}$$

The steady state kinetic law for this reaction scheme can be straightforwardly obtained by equating right-hand sides of equation (A.1) to zero and using normalization condition *e1+e2+e3+e4*= 1 to express enzyme probabilities as a function of [*S*] and [*I*]. Using the results in equation (A.2) leads to a slightly cumbersome rate expression is given by[1,3]

$$v = \frac{V}{[E]_T} = \frac{\kappa_{21}[S]\left(K_1[S]+K_2[I]+K_3\right)+\kappa_{43}[I][S]\left(K_4[S]+K_5[I]+K_6\right)}{K_7+K_8[S]+K_9[I]+K_{10}[S][I]+K_1[S]^2+K_{11}[I]^2+K_5[I]^2[S]+K_4[S]^2[I]} \qquad \text{(A.3)}$$

where K_1 to K_{11} are combination of rate constants

$$K_1 = k_{12}k_{42}k_{34},\ K_2 = k_{13}k_{42}k_{34},\quad K_3 = k_{12}k_{31}\left(k_{42} + k_{43} + \kappa_{43}\right),\ K_4 = k_{12}k_{24}k_{34},\quad K_5 = k_{13}k_{24}k_{34},$$

$$K_6 = \frac{k_{12}k_{24}k_{31}}{k_{21}k_{42}}\left(k_{21}k_{42} + k_{21}k_{43} + \kappa_{21}k_{43}\right),\quad K_7 = k_{31}\left(k_{21} + \kappa_{21}\right)\left(k_{42} + k_{43} + \kappa_{43}\right),$$

$$K_8 = k_{42}k_{34}\left(k_{21} + \kappa_{21}\right) + k_{12}k_{31}\left(k_{42} + k_{43} + \kappa_{43}\right), K_9 = \frac{k_{12}k_{24}k_{43}k_{31}}{k_{21}k_{42}k_{34}}\left(k_{21} + \kappa_{21}\right) + \frac{k_{21}k_{42}k_{34}k_{13}}{k_{12}k_{43}}\left(k_{43} + \kappa_{43}\right)$$

$$K_{10} = \frac{k_{12}k_{24}k_{31}}{k_{21}k_{42}}\left(k_{21}k_{42} + k_{43}k_{21} + k_{42}\kappa_{21}\right) + k_{12}k_{34}\left(k_{43} + \kappa_{43}\right) + k_{42}k_{13}k_{43},\quad K_{11} = k_{13}k_{24}\left(k_{43} + \kappa_{43}\right)$$

In the quasi-equilibrium limit κ_{21} and κ_{43} are very small. Using the detailed balance condition ($k_{12}k_{24}k_{43}k_{31} = k_{21}k_{42}k_{34}k_{13}$), the complete velocity equation is simplified to:

$$v = \frac{V}{[E]_T} = \frac{\kappa_{21}k_{12}k_{42}k_{34}[S] + \kappa_{43}k_{12}k_{24}k_{34}[S][I]}{k_{21}k_{42}k_{34} + k_{12}k_{42}k_{34}[S] + k_{12}k_{24}k_{43}[I] + k_{12}k_{24}k_{34}[S][I]}\quad . \tag{A.4}$$

This is the kinetic law in the quasi-equilibrium limit.

Using the same approach for the kinetic scheme shown in Fig. 1b (bisubstrate random order reaction with the catalytic rate κ_{41} smaller than k_{34} and k_{24}) we obtain in quasi- equilibrium limit

$$v = \frac{V}{[E]_T} = \frac{\kappa_{41}k_{12}k_{24}k_{34}[S_1][S_2]}{k_{21}k_{42}k_{34} + k_{12}k_{42}k_{34}[S_1] + k_{12}k_{24}k_{43}[S_2] + k_{12}k_{24}k_{34}[S_1][S_2]} \tag{A.5}$$

B: Conformational dynamics for random-order bisubstrate and partial noncompetitive inhibition reactions

Random order bisubstrate reaction

Let's consider the reaction scheme Fig. 1b: bi-substrate random order reaction. The complete velocity equation for this reaction scheme in the quasi- equilibrium limit is given by equation (A.5). When the transition rates are dependent on the conformational coordinate x of the enzyme,

the enzyme concentrations are replaced by their steady state distributions, which can be obtained by solving the reaction diffusion equations (equation (17)) which in this case take the form

$$\begin{aligned}
&\left(L_1(x)-\left(k_{12}(x)[S_1]+k_{13}(x)[S_2]\right)\right)P_1^{ss}(x)+k_{21}(x)P_2^{ss}+k_{31}(x)P_3^{ss}(x)+\kappa_{41}(x)P_4^{ss}(x)=0\\
&\left(L_2(x)-\left(k_{21}(x)+k_{24}(x)[S_2]\right)\right)P_2^{ss}(x)+k_{12}(x)[S_1]P_1^{ss}(x)+k_{42}(x)P_4^{ss}(x)=0\\
&\left(L_3(x)-\left(k_{34}(x)[S_1]+k_{31}(x)\right)\right)P_3^{ss}(x)+k_{13}(x)[S_2]P_1^{ss}(x)+k_{43}(x)P_4^{ss}(x)=0\\
&\left(L_4(x)-\left(k_{43}(x)+k_{42}(x)+\kappa_{41}(x)\right)\right)P_4^{ss}(x)+k_{24}(x)[S_2]P_2^{ss}(x)+k_{34}(x)[S_1]P_3^{ss}(x)=0
\end{aligned} \tag{B.1}$$

Using the ansatz given in equation (21) in equation (45) for our reaction scheme, we have

$$v=\frac{\int \kappa_{41}(x)P_4^0(x)e^{-\beta U_{ss}(x)}dx}{\int e^{-\beta U_{ss}(x)}dx} \tag{B.2}$$

In the quasi-equilibrium limit, using equation (21) and (22), the coupled diffusion equations in equation (B.1) without diffusion terms can be solved for the local steady state probabilities $P_j^0(x)$. Using the detailed balance condition for the closed loop in Fig. 1b ($k_{12}k_{24}k_{43}k_{31}=k_{21}k_{42}k_{34}k_{13}$), together with equation (35) we have

$$\begin{aligned}
P_1^0(x)&=\frac{C_1e^{-\beta U_1(x)}}{C_1e^{-\beta U_1(x)}+C_2e^{-\beta U_{21}(x)}+C_3e^{-\beta U_3(x)}+C_4e^{-U_4(x)}}\\
P_2^0(x)&=\frac{C_2e^{-\beta U_2(x)}}{C_1e^{-\beta U_1(x)}+C_2e^{-\beta U_2(x)}+C_3e^{-\beta U_3(x)}+C_4e^{-U_4(x)}}\\
P_3^0(x)&=\frac{C_3e^{-\beta U_3(x)}}{C_1e^{-\beta U_1(x)}+C_2e^{-\beta U_2(x)}+C_3e^{-\beta U_3(x)}+C_4e^{-U_4(x)}}\\
P_4^0(x)&=\frac{C_4e^{-U_4(x)}}{C_1e^{-\beta U_1(x)}+C_2e^{-\beta U_2(x)}+C_3e^{-\beta U_3(x)}+C_4e^{-U_4(x)}}
\end{aligned} \tag{B.3}$$

where $C_1=k_{21}^0k_{34}^0k_{42}^0$, $C_2=k_{12}^0k_{34}^0k_{42}^0[S_1]$, $C_3=k_{12}^0k_{24}^0k_{43}^0[S_2]$, and $C_4=k_{12}^0k_{24}^0k_{34}^0[S_1][S_2]$

Using the definitions of C_1, C_2, C_3, C_4, equation (16) and (40), the steady state velocity equation in equation (B.2) reduces to

$$v=\frac{\langle\kappa_{41}\rangle_4\, k_{12}^0k_{24}^0k_{34}^0[S_1][S_2]}{k_{21}^0k_{34}^0k_{42}^0+k_{12}^0k_{34}^0k_{42}^0[S_1]+k_{12}^0k_{24}^0k_{43}^0[S_2]+k_{12}^0k_{24}^0k_{34}^0[S_1][S_2]} \tag{B.4}$$

where $\langle\kappa_{41}\rangle_4=\int\kappa_{41}(x)e^{-\beta U_4(x)}$

Equation (B.4) has the same structural form as equation (A.5) with position-independent prefactors used as rates.

Partial noncompetitive inhibition mechanism

Let us consider the reaction scheme described in Fig. 1c. The complete velocity equation for this reaction scheme in the quasi-equilibrium limit is given by equation (A.4). When the transition rates are dependent on the conformational coordinate of the enzyme, one can get the four linearly dependent reaction diffusion equations for the steady state distributions as shown in equation (17) which is given by

$$\begin{aligned}
&\left(L_1(x)-\left(k_{12}(x)[S]+k_{13}(x)[I]\right)\right)P_1^{ss}(x)+\left(k_{21}(x)+\kappa_{21}(x)\right)P_2^{ss}+k_{31}(x)P_3^{ss}(x)=0\\
&\left(L_2(x)-\left(k_{21}(x)+k_{24}(x)[I]+\kappa_{21}(x)\right)\right)P_2^{ss}(x)+k_{12}(x)[S]P_1^{ss}(x)+k_{42}(x)P_4^{ss}(x)=0\\
&\left(L_3(x)-\left(k_{34}(x)[S]+k_{31}(x)\right)\right)P_3^{ss}(x)+k_{13}(x)[I]P_1^{ss}(x)+\left(k_{43}(x)+\kappa_{41}(x)\right)P_4^{ss}(x)=0\\
&\left(L_4(x)-\left(k_{43}(x)+k_{42}(x)+\kappa_{41}(x)\right)\right)P_4^{ss}(x)+k_{24}(x)[I]P_2^{ss}(x)+k_{34}(x)[S]P_3^{ss}(x)=0
\end{aligned} \tag{B.5}$$

Applying the *ansatz* in equation (21) into equation (47), we have

$$v=\frac{\int\left(\kappa_{21}(x)P_2^0(x)+\kappa_{43}(x)P_4^0(x)\right)e^{-\beta U_{ss}(x)}dx}{\int e^{-\beta U_{ss}(x)}dx} \tag{B.6}$$

In the quasi-equilibrium limit (when κ_{21} and κ_{43} are very small), the diffusion equations can be solved for local steady state probabilities ($P_i^0(x), i=1,2,3,4$) using the *ansatz* in equation (21) and can be simplified further using the detailed balance condition for reversible binding and dissociation

Using these reduced expressions for the local steady state probabilities, the steady state velocity equation reduce to

$$v=\frac{\langle\kappa_{21}\rangle_2 k_{12}^0 k_{42}^0 k_{34}^0[S]+\langle\kappa_{43}\rangle_4 k_{12}^0 k_{24}^0 k_{34}^0[S][I]}{k_{21}^0 k_{34}^0 k_{42}^0+k_{12}^0 k_{34}^0 k_{42}^0[S]+k_{12}^0 k_{24}^0 k_{43}^0[I]+k_{12}^0 k_{24}^0 k_{34}^0[S][I]} \quad \text{(B.7)}$$

where $\langle\kappa_{21}\rangle_2=\int\kappa_{21}(x)e^{-\beta U_2(x)}$ and $\langle\kappa_{43}\rangle_4=\int\kappa_{43}(x)e^{-\beta U_4(x)}$

equation (B.7) has the same dependence on the substrate and inhibitor concentration as obtained from conventional mass action kinetics (equation (A.4)) with position-independent prefactors used as rates.

C: Decoupling ansatz is exact in quasi-equilibrium limit

Under the quasi-equilibrium condition, the catalytic rates are much slower than other transitions. In this limit the equations for the steady state distributions and the equilibrium distributions are identical. Thus equation (17) that determines the steady state distributions reduces to:

$$L_i(x)P_i^{ss}(x)-\sum_{j=1}^{N}k_{ij}(x)P_i^{ss}(x)+\sum_{j=1}^{N}k_{ji}(x)P_j^{ss}(x)=0 \quad \text{(C.1)}$$

Using the detailed balance condition we conclude that the fluxes of individual reversible reactions are zero, i.e.

$$k_{ij}(x)P_i^{ss}(x)=k_{ji}(x)P_j^{ss}(x) \quad \text{(C.2)}$$

Using equation (34), equation (C.2) can be written as

$$\frac{P_j^{ss}(x)}{P_j^{ss}(x)}=\frac{k_{ij}(x)}{k_{ji}(x)}=\frac{k_{ij}^0}{k_{ji}^0}\exp\left\{\beta\left[U_i(x)-U_j(x)\right]\right\} \quad \text{(C.3)}$$

Here we consider a trial solution for $P_j^{ss}(x)$ which is given by

$$P_j^{ss}(x)=F_j(x)e^{-\beta U_j(x)} \quad \text{(C.4)}$$

Using equation (C.4) in equation (C.3) we obtain

$$\frac{F_j(x)}{F_i(x)} = \frac{k_{ij}^0 C_i}{k_{ji}^0 C_j}$$

Hence all $F_j(x)$ have the same dependence on conformational coordinate, i.e. $F_j(x) = F(x) A_j$

Using this relation in equation (C.4), we obtain

$$P_j^{ss}(x) = F(x) A_j e^{-\beta U_j(x)} \tag{C.5}$$

This form is the same as the *ansatz* in equation (21) if one takes into account equation (38). Thus we find that this *ansatz* in equation (21) is exact in the quasi-equilibrium limit.

D: Discrete-state model for conformational fluctuations in the quasi-equilibrium limit

In the main text we have considered the effect of conformational fluctuations in the quasi-equilibrium limit. Our results showed that in this limit, together with the detailed balance condition, the steady state rate law has the same dependence on the substrate concentration as in mass action kinetics and is independent of the conformational dynamics of the different states. Here we further illustrate this result by considering a discretized model for a simple enzyme catalyzed reaction.

We consider two enzyme states E_1 and E_2 that combine reversibly with the substrate S to give the enzyme-substrate complex ES_1 and ES_2 respectively. These two interconverting enzyme-substrate complexes dissociate to give the product P. The full kinetic scheme describing the discrete state approximation of our continuum model that we discuss in the main text is given in the following reaction scheme

$$
\begin{aligned}
&E_1 + S \underset{k_{1b}}{\overset{k_{1f}}{\rightleftharpoons}} ES_1 \xrightarrow{k_{cat1}} E_1 + P \\
&E_2 + S \underset{k_{2b}}{\overset{k_{2f}}{\rightleftharpoons}} ES_2 \xrightarrow{k_{cat2}} E_2 + P \\
&E_1 \underset{a_2}{\overset{a_1}{\rightleftharpoons}} E_2 \\
&ES_1 \underset{b_2}{\overset{b_1}{\rightleftharpoons}} ES_2
\end{aligned} \tag{D.1}
$$

The detailed balance condition for reversible binding is given as

$$
k_{1f} b_1 k_{2b} a_2 = k_{2f} b_2 k_{1b} a_1 \tag{D.2}
$$

Starting from the formalism proposed by Gopich and Szabo[14], we define a matrix ***K***, that describes transitions between different states including conformational changes. The steady state probability $P_{ss}(i)$ of finding the system in the state i can be written in the form of the following matrix equation

$$
\begin{aligned}
&KP_{ss} = 0 \\
&1^{\dagger} P_{ss} = 1
\end{aligned} \tag{D.3}
$$

1 is the unit vector and † denotes transpose. We can apply this formalism to enzymatic reactions scheme presented in equation A.1.

For this the matrix **K** in the basis (E_1, E_2, ES_1, ES_2) is given by

$$
\mathbf{K} = \begin{pmatrix}
-\left(k_{1f}\left[S\right] + a_1\right) & a_2 & k_{1b} + k_{cat1} & 0 \\
a_1 & -\left(k_{2f} + a_2\right) & 0 & k_{2b} + k_{cat2} \\
k_{1f}\left[S\right] & 0 & -\left(k_{1b} + k_{cat1} + b_1\right) & b_2 \\
0 & k_{2f} & b_1 & -\left(k_{2b} + k_{cat2} + b_2\right)
\end{pmatrix} \tag{D.4}
$$

The steady state rate can be expressed as

$$k = k_{cat1} P_{ss}(ES_1) + k_{cat2} P_{ss}(ES_2) \tag{D.5}$$

$P_{ss}(ES_1)$ and $P_{ss}(ES_2)$ are the steady state probabilities and can be obtained by using Equation (D.3) and (D.4)

In the quasi-equilibrium limit, the catalytic rates k_{cat1} and k_{cat2} are small and can be expressed as

$k_{cat1} = k_1 x$ and $k_{cat2} = k_2 x$. Taking the limit of very small x and applying the detailed balance condition given in Equation (D.2), the steady state rate can be written as

$$k = \frac{k_{1f}\left(\dfrac{b_2 k_1}{b_1} + k_2\right) S}{\left(\dfrac{b_2 k_{1b} k_{2f}}{b_1} + k_{1f} k_{2b}\right) + k_{1f} k_{2f} S \left(\dfrac{b_2}{b_1} + 1\right)} \tag{D.6}$$

The steady state rate is independent of the interconversion rates a_1 and a_2 and depends on the ration of b_1/b_2 which in turn is independent of the rate of conformational transitions, i.e. the diffusion constants in the continuous formalism used in the main text.
